# β1 Integrin as a Prognostic and Predictive Marker in Triple-Negative Breast Cancer

**DOI:** 10.3390/ijms17091432

**Published:** 2016-08-31

**Authors:** Hsin-Ling Yin, Chun-Chieh Wu, Chih-Hung Lin, Chee-Yin Chai, Ming-Feng Hou, Shu-Jyuan Chang, Hung-Pei Tsai, Wen-Chun Hung, Mei-Ren Pan, Chi-Wen Luo

**Affiliations:** 1 Department of Pathology, Kaohsiung Medical University Hospital, Kaohsiung Medical University, 807 Kaohsiung, Taiwan; fp7081@gmail.com (H.-L.Y.); lazzz_wu@yahoo.com.tw (C.-C.W.); chlathelas@gmail.com (C.-H.L.); cychai@kmu.edu.tw (C.-Y.C.); 2 Department of Pathology, Faculty of Medicine, Collage of Medicine, Kaohsiung Medical University, 807 Kaohsiung, Taiwan; 3 Graduate Institute of Medicine, College of Medicine, Kaohsiung Medical University, 807 Kaohsiung, Taiwan; mifeho@kmu.edu.tw (M.-F.H.); binbin4728@hotmail.com (S.-J.C.); carbugino@gmail.com (H.-P.T.); 4 Cancer Center, Kaohsiung Medical University Hospital, 807 Kaohsiung, Taiwan; hung1228@nhri.org.tw; 5 Graduate Institute of Clinical Medicine, Kaohsiung Medical University, 807 Kaohsiung, Taiwan; 6 National Institute of Cancer Research, National Health Research Institutes, 704 Tainan, Taiwan; 7 Research Center for Environmental Medicine, Kaohsiung Medical University, 807 Kaohsiung, Taiwan

**Keywords:** Triple negative breast cancer, β1 integrin, migration, invasion, drug sensitivity

## Abstract

Triple negative breast cancer (TNBC) displays higher risk of recurrence and distant metastasis. Due to absence of estrogen receptor (ER), progesterone receptor (PR) and human epidermal growth factor receptor 2 (HER2), TNBC lacks clinically established targeted therapies. Therefore, understanding of the mechanism underlying the aggressive behaviors of TNBC is required for the design of individualized strategies and the elongation of overall survival duration. Here, we supported a positive correlation between β1 integrin and malignant behaviors such as cell migration, invasion, and drug resistance. We found that silencing of β1 integrin inhibited cell migration, invasion, and increased the sensitivity to anti-cancer drug. In contrast, activation of β1 integrin increased cell migration, invasion, and decreased the sensitivity to anti-cancer drug. Furthermore, we found that silencing of β1 integrin abolished Focal adhesion kinese (FAK) mediated cell survival. Overexpression of FAK could restore cisplatin-induced apoptosis in β1 integrin-depleted cells. Consistent to in vitro data, β1 integrin expression was also positively correlated with FAK (*p* = 0.031) in clinical tissue. More importantly, β1 integrin expression was significantly correlated with patient outcome. In summary, our study indicated that β1 integrin could regulate TNBC cells migration, invasion, drug sensitivity, and be a potential prognostic biomarker in TNBC patient survival.

## 1. Introduction

Triple negative breast cancer (TNBC) is a specific breast cancer subtype that is immunohistochemically negative for expressions of prognostic markers including estrogen receptor (ER), progesterone receptor (PR), and human epidermal growth factor receptor 2 (HER2) [1]. TNBC accounts for 10%–20% of all breast cancers, more frequently arises in younger patients, and is more prevalent in African-American women [2]. Compared to other breast tumors, TNBC tumors are generally more aggressive and have larger size and higher grade [3]. Additionally, TNBC patients often present with early distant metastases and lymph node involvement at the time of diagnosis [3,4]. This aggressive metastatic phenomenon contributes to short overall survival in patients with TNBC [5,6]. Paradoxically, despite TNBC patients having higher response rates to presurgical (neoadjuvant) chemotherapy, these patients still have higher rates of distant metastasis and poorer prognosis than do patients with other breast cancer subtypes [3,7]. The dissemination of cancer cells from the primary tumor to a distant organ is the most common cause of death for patients with cancer [8]. Therefore, how to decrease the rate of metastasis in TNBC patients is an important issue.

Integrins, which are transmembrane receptors in a large family of 18α and 8β heterodimeric transmembrane proteins, were well known as adhesion molecules in mediating cell-extracellular matrix (ECM) interaction [9]. Integrins are important sensors of the cell microenvironment and regulate many intracellular and extracellular signaling pathways involving the organization of cells, tissues and organs during development in response to structural variations of the extracellular matrix. Integrins have been implicated in many processes associated with tumor cell adhesion to the extracellular matrix (ECM), including migration, invasion, and metastasis [10,11,12]. Integrins also play significant roles in regulating cell apoptosis-associated gene expression [13]. Because of their critical roles in many cellular processes that are hallmarks of cancer development, integrins are also important therapeutic targets [14].

Recent attention has been focused on the role of β1 integrin in malignant phenotype [15]. Previous studies have indicated that β1 integrin is an important integrin expressed in normal cells and in tumor-associated cells since it controls various development processes [16,17,18,19,20,21,22,23]. Other studies also show that, in many cancer types, β1 integrin might induce resistance to radiotherapies, chemotherapies, and target therapies [24,25,26,27,28]. Noted, several β1 integrin antagonists also have been studied in recent years [29,30]. Based on these findings, β1 integrin could be applied to several types of cancers including breast cancer [31]. However, whether β1 integrin plays an important role in mediating the migration and invasion capacities and drug resistance in TNBC cells is still unclear. In current study, we investigated the roles of β1 integrin in three TNBC cells (MDA-MB-231, HS-578T, and MDA-MB-468) and TNBC patients. Our results indicated that β1 integrin could regulate the migration, invasion, and epithelial-mesenchymal transition (EMT) in TNBC cells. The survival rates of cells after cisplatin treatment were clearly decreased in β1 integrin knockdown cells but restored in β1 integrin-activating cells. Clinical analyses also showed that β1 integrin was related to metastasis, recurrence, and survival in TNBC patients. Thus, our study provides convincing evidence that β1 integrin is a potential prognostic biomarker for TNBC patients.

## 2. Results

### 2.1. Expression of β1 Integrin in Normal Mammary Epithelial Cells and Triple Negative Breast Cancer (TNBC) Cells

**Figure 1:**
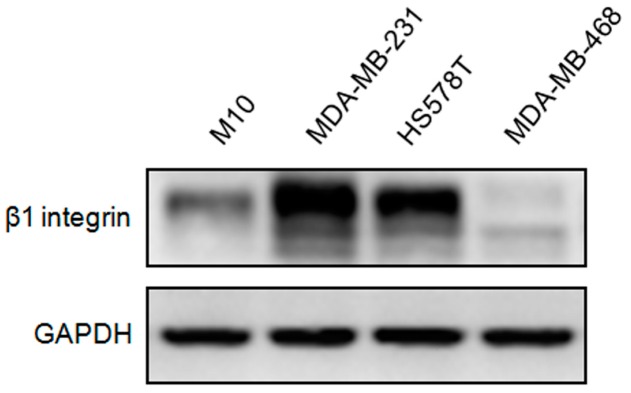
Comparison of β1 integrin expression between normal and Triple negative breast cancer (TNBC) cells. Expression of β1 integrin was compared in normal human mammary epithelial cell (M10) and three TNBC cell lines (MDA-MB-231, HS578T and MDA-MB-468). Cell lysates were immunoblotted by anti-β1 integrin. Glyceraldhyde-3-phosphate dehydrogenase (GAPDH) was used as a loading control. The experiments were performed independently at least three times.

To analyze the role of β1 integrin on TNBC cells, we first compared the basal expression of β1 integrin in normal human mammary epithelial cell (M10) and three TNBC cells (MDA-MB-231, MDA-MB-468 and HS578T). Figure 1 shows the immunoblot results, which indicated that MDA-MB-231 and HS578T cells had higher expression of β1 integrin compared to M10 and MDA-MB-468 cells. In this series of experiments, MDA-MB-231 and HS578T cells were transfected with short interfering Control (wild type, WT) and β1 integrin silencing (KO), and MDA-MB-468 was incubated with mouse IgG1 negative control (wild type, WT) and β1 integrin activator (MAB).

### 2.2. β1 Integrin Affected TNBC Cell Migration and Invasion

**Figure 2:**
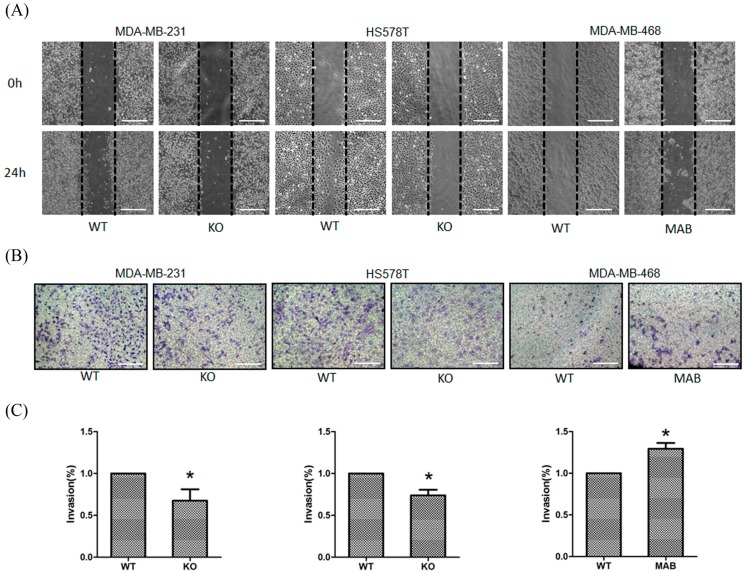
Validation the roles of β1 integrin in TNBC cell migration and invasion. (**A**) Parental (WT) and β1 integrin silencing (KO) MDA-MB-231 and HS578T cells, parental (WT) and β1 integrin activating (MAB) MDA-MB-468 cell were cultured until reaching 90% confluence. A scratch was made with pipette in each well. Cell migration was photographed at 0 h and 24 h. Magnification ×200, scale bar: 100 μm. The experiments were performed independently for at least three times; (**B**) MDA-MB-231 and HS578T cells were transfected with control and β1 integrin siRNA for 24 h. MDA-MB-468 cell was treated with IgG control and β1 integrin activator for 6 h. Cells were analyzed by transwell cell culture chambers after 24 h. Cell invasion was photographed at 24 h. Magnification: ×400, scale bar: 50 μm; (**C**) Bar chart showing the quantification of cell invasion by counting the number of cell invade underside of the membrane. Values represent the mean ± SD from at least 3 experiments. Statistically significant data are indicated by * for significant at *p* < 0.05.

The effects of β1 integrin on TNBC cells motility were investigated by using a standard wound-healing assay. As shown in Figure 2A, knockdown of β1 integrin significantly led to the reduction of migration in MDA-MB-231 and HS578T cells compared to parental cells. A transwell invasion assay was then used to study invasion ability in both cell lines. As shown in Figure 2B,C, invasion ability was suppressed in β1 integrin knockdown cells. In contrast, activation of β1 integrin increased migration and invasion in MDA-MB-468 cells (Figure 2A–C). Hence, these results suggested that β1 integrin affected the migration and invasion of TNBC cells.

### 2.3. β1 Integrin Regulated Expressions of Proteins Related to the Epithelial-Mesenchymal Transition (EMT) in TNBC Cells

**Figure 3:**
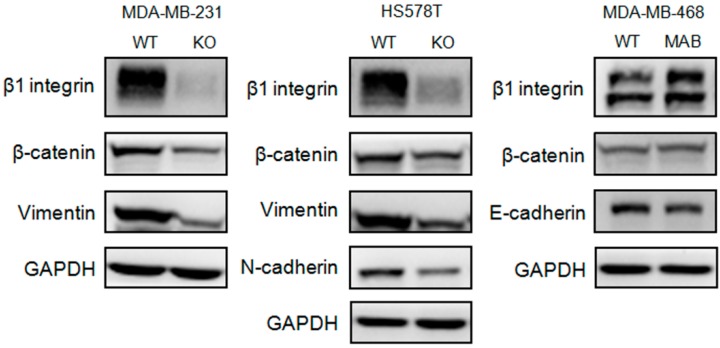
β1 integrin regulates the epithelial-mesenchymal transition (EMT) relative protein expressions in TNBC cells. Cell lysates from parental and β1 integrin silencing MDA-MB-231 and HS578T cells, and parental and β1 integrin activating MDA-MB-468 cell were immunoblotted by anti-β1 integrin, anti-β catenin, anti-N-cadherin, anti-E-cadherin and anti-Vimentin. GAPDH was used as a loading control. Data were collected in at least three independent experiments.

To further verify the role of β1 integrin in contributing cell mobility, we next determined the role of β1 integrin in the process of EMT. As performed in Figure 3, we found that the depletion of β1 integrin decreased a series of EMT-associated proteins including N-cadherin, β-catenin, and vimentin in MDA-MB-231 and HS578T cells. Conversely, activation of β1 integrin increased the level of β-catenin and decreased the level of E-cadherin in MDA-MB-468 cells. These experimental results strongly indicated that levels of β1 integrin could alter several proteins related to the EMT in TNBC cells.

### 2.4. Inhibition of β1 Integrin Attenuated Cisplatin-Induced Apoptosis

**Figure 4:**
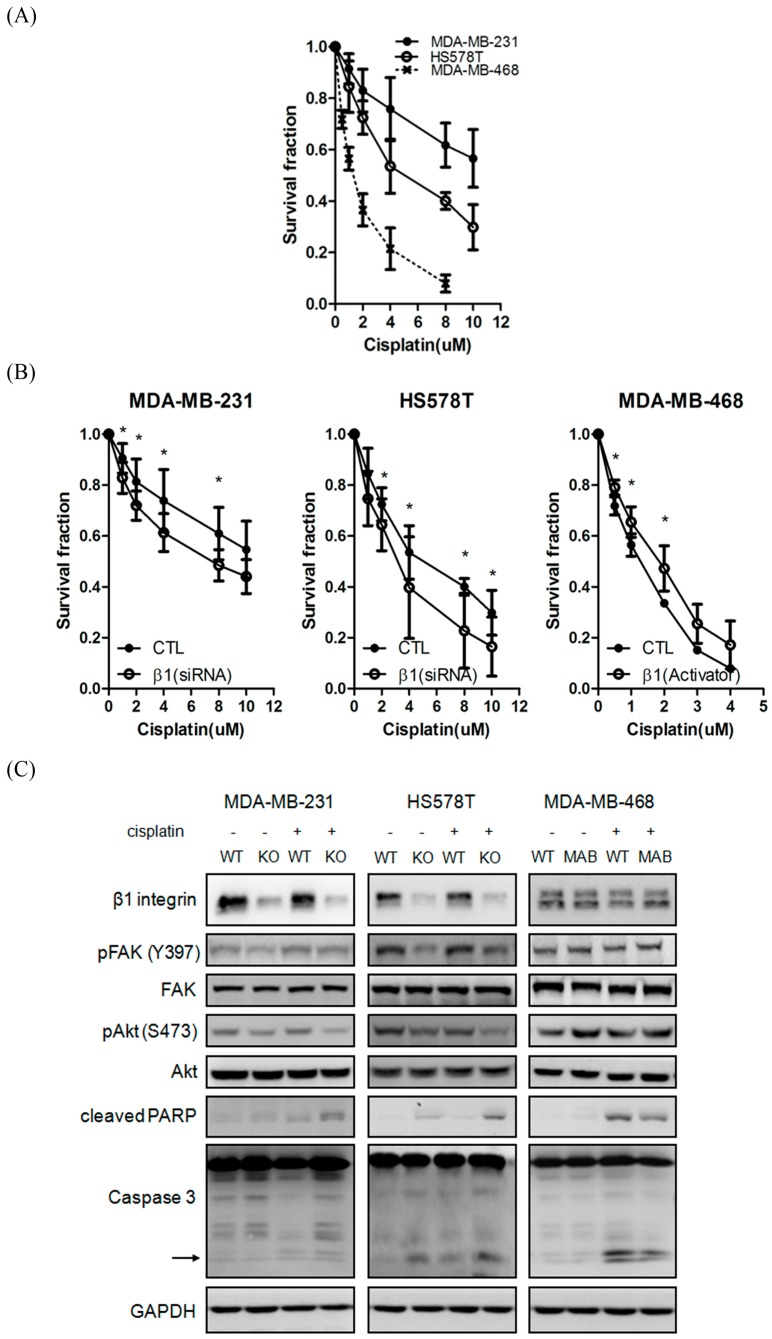
β1 integrin regulates cisplatin sensitivity in TNBC cells. (**A**) 3-(4,5-Dimethylthiazol-2-yl)-2,5-Diphenyltetrazolium bromide (MTT) assay showed the cisplatin sensitivity in parental MDA-MB-231, HS578T, and MDA-MB-468 cells; (**B**) MDA-MB-231 and HS578T cells were transfected with control and β1 integrin siRNA for 24 h, and MDA-MB-468 cell was treated with IgG control and β1 integrin activator for 6 h. After silencing or activating the β1 integrin, cells were treated with different doses of cisplatin. MTT assay showed that the β1 integrin silencing enhanced the toxic effect of cisplatin in MDA-MB-231 and HS578T cells, whereas β1 integrin activation inhibited the toxic effect of cisplatin in MDA-MB-468 cells. Values are represented as mean ± SD in at least three independent experiments. Statistically significant data are indicated by * for significant at *p* < 0.05; (**C**) Silencing or activating of β1 integrin regulated TNBC cell death through apoptotic pathway. Cell lysates were immunoblotted by anti-β1 integrin, anti-cleaved PARP, and anti-Caspase3. GAPDH was used as a loading control. Data were collected in at least three independent experiments.

In order to further clarify the role of β1 integrin in TNBC cells, we next examined if β1 integrin plays a regulatory role in the drug sensitivity of TNBC cells. Figure 4A shows the cisplatin sensitivity in three different TNBC cells. MDA-MB-231 and HS578T cells were more resistant to cisplatin than MDA-MB-468 cell. We found that knockdown of β1 integrin increased cisplatin sensitivity in MDA-MB-231 and HS578T cells. Conversely, activation of β1 integrin can efficiently decrease the toxic effect of cisplatin to MDA-MB-468 cell (Figure 4B). Previous data reported that cisplatin damages tumors via induction of apoptosis. We next determined whether β1 integrin played a role in mediating cisplatin-induced apoptosis. Consistent to our hypothesis, knockdown of β1 integrin increased cleaved forms of PARP and caspase 3 in both cells, as performed by western blot. It indicated that β1 integrin defects triggered the increase of toxic effect of cisplatin via induction of the apoptotic pathway (Figure 4C).

### 2.5. β1 Integrin Modulates FAK-Mediated Drug Sensitivity and Cell Survival 

**Figure 5:**
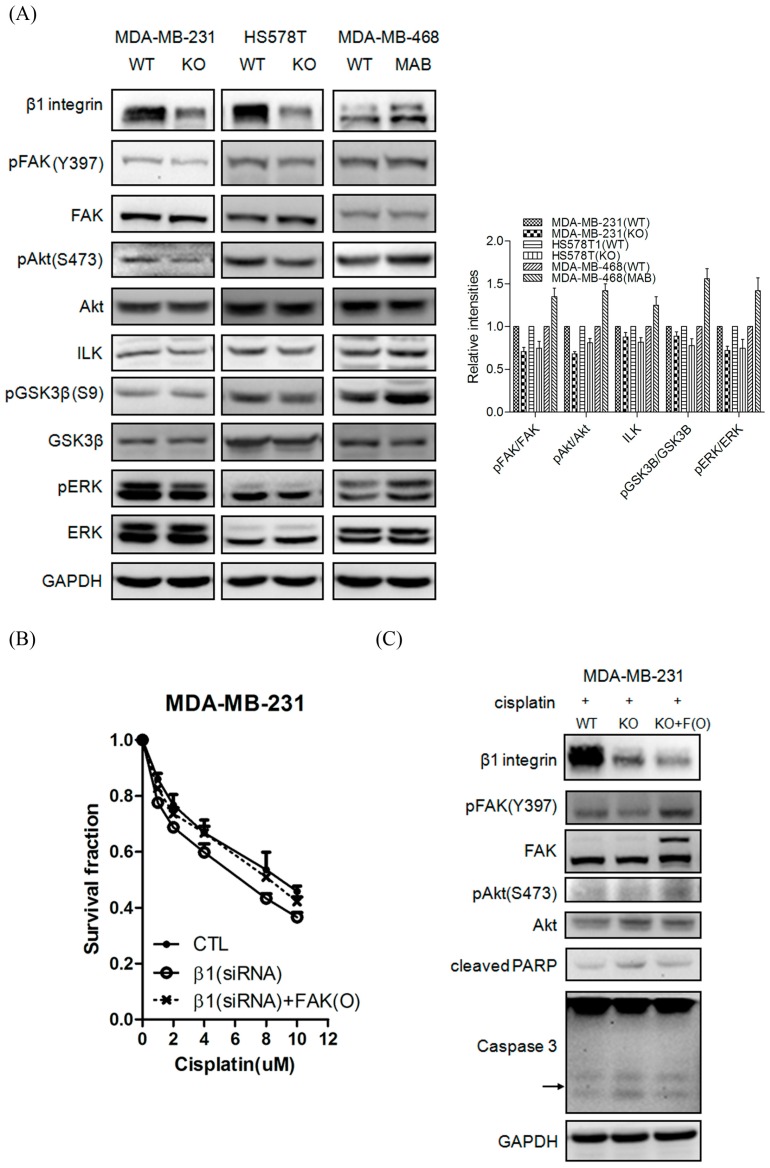
β1 integrin modulates FAK-mediated drug sensitivity and cell survival. (**A**) MDA-MB-231 and HS578T cells were transfected with control and β1 integrin siRNA for 24 h and MDA-MB-468 cell was treated with IgG control and β1 integrin activator for 6 h. Cell lysates were immunoblotted by anti-β1 integrin, anti-FAK, anti-pFAK (Y397), anti-Akt, anti-pAkt (s473), anti-ERK, anti-pERK, anti-ILK, anti-GSK3β, and anti-p-GSK3β (ser9), GAPDH was used as a loading control. Data were collected in at least three independent experiments; (**B**) MTT assay showed that overexpression of FAK can overcome the cisplatin-mediating cytotoxic effect in β1 integrin defects cells; (**C**) Western blot data showed the enhanced of cytotoxicity of cisplatin in β1 integrin silencing (KO) cells were restored by overexpression of FAK (KO+F(O)). Cell lysates were immunoblotted by anti-β1 integrin, anti-pFAK, anti-FAK, anti-pAkt, anti-Akt, anti-cleaved PARP, and anti-Caspase3. GAPDH was used as a loading control. Data were collected in at least three independent experiments.

To further characterize the details of the mechanism in β1 integrin-mediating drug sensitivity, we next sought to identify the downstream signal pathway involved in TNBC. As shown in Figure 5A, knockdown of β1 integrin resulted in decreasing phosphorylated levels of FAK and Protein kinase B (Akt) in MDA-MB-231 and HS578T cells, compared to WT cells in our experiments. In addition, ILK and phosphorylated levels of Extracellular signal-regulated kinase (ERK) and GSK3β also decreased after knockdown of β1 integrin. In contrast, activation of β1 integrin in MDA-MB-468 cells resulted in increasing the levels of phosphorylated FAK, Akt and ERK compared to parental cells (Figure 5A). Importantly, our MTT assay and western blot results indicated that overexpression of FAK could dramatically overcome the cisplatin-mediating cytotoxic effect in β1 integrin defects cells (Figure 5B,C). In summary, β1 integrin-mediated regulation of FAK-Akt signals in contributing drug sensitivity and migration ability.

### 2.6. Relationships Between β1 Integrin and Clinicopathological Parameters

**Figure 6:**
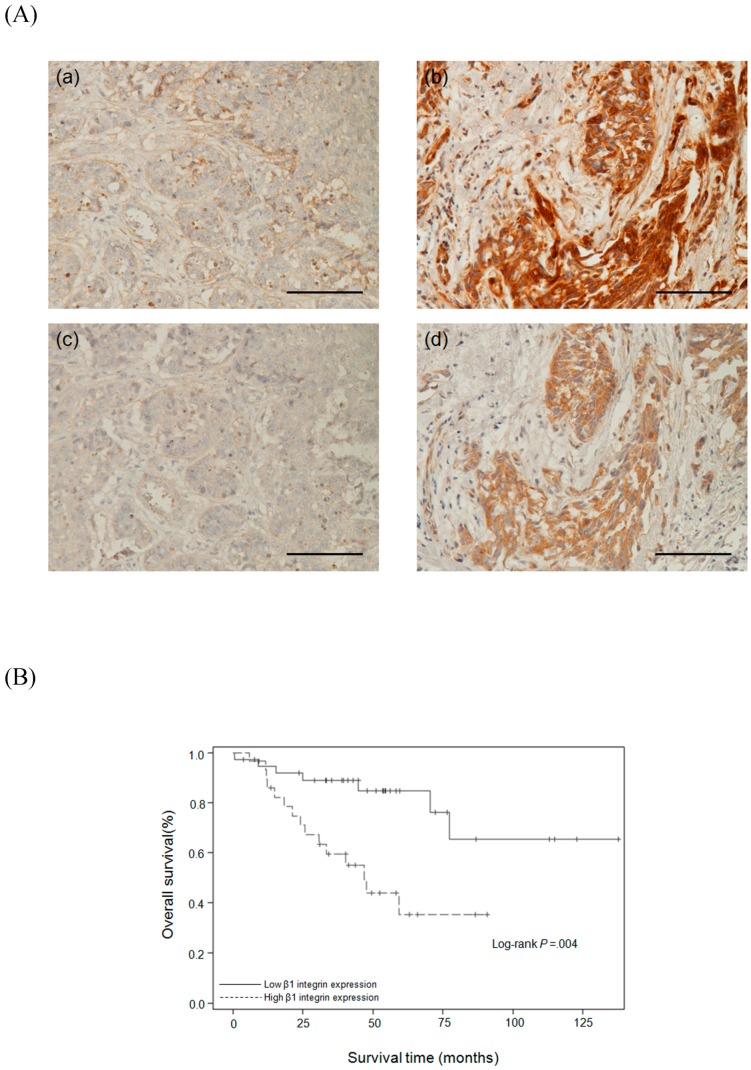
Evaluation of β1 integrin as a prognosis marker in TNBC patients. (**A**) Representative immunostaining results for expressions of β1 integrin and FAK in TNBC tissues (original magnification: ×200, scale bar: 100μm). Immunoreactivity of β1 integrin and FAK were classified as negative ((**a**) and (**c**), respectively) or positive ((**b**) and (**d**), respectively) according to staining observed in the cell membrane and cytoplasm; (**B**) Kaplan-Meier survival curves for TNBC patients. Survival was significantly associated with β1 integrin expression.

High expression of β1 integrin has been reported in many cancer types but is still unclear in TNBC. In order to confirm our in vitro data, immunohistochemical staining was used to investigate the relationships between β1 integrin and other clinicopathological parameters in biopsy specimens from 67 TNBC patients. The patients had an average age of 51 ± 10 years (range 32–81 years) and an average tumor size of 2.5 ± 1.5 cm (range 0.13–7.5 cm). Figure 6A shows the TNBC patients with low expression ((a) and (c) respectively) and high expression ((b) and (d) respectively) of β1 integrin and FAK. Consistent with the in vitro data, there was a positive correlation between β1 integrin and FAK (Table 1). Table 2 indicates that β1 integrin was significantly associated with metastasis, recurrence, and death (*p* = 0.039, 0.038, and 0.004 respectively).

### 2.7. Survival Analysis

We further investigated whether β1 integrin expression was associated with patient survival. The Kaplan-Meier test was used to compare the survival time between patients with high and low β1 integrin expression. Figure 6B shows that the average disease-specific survival time (months from time of TNBC diagnosis to time of death due to TNBC) in patients with high β1 integrin expression was 47.0 ± 30.9 months (range, 0.53–137.7 months), which was significantly lower than that in patients with low β1 integrin expression (*p* = 0.002). The univariate analysis (Table 3) revealed that overall survival was significantly associated with β1 integrin expression (*p* = 0.0004), nodal stage (*p* < 0.0001), metastatic stage (*p* = 0.0019), and tumor recurrence (*p* = 0.0436). Multivariate Cox regression analysis of β1 integrin expression, age, tumor grade, nodal stage, metastatic stage, and tumor recurrence showed that β1 integrin expression was a significant independent predictor of overall survival (*p* = 0.0476). Taken together, the experimental data indicate that β1 integrin has potential use as biomarker of TNBC survival and as a biomarker of TNBC cell migration, invasion, and drug resistance.

## 3. Discussion

High level of β1 integrin has been associated with poor outcomes in many types of tumors including colon cancer, pancreatic cancer, lung cancer, ovarian cancer and breast cancer [32]. Previous studies have indicated that the increase of β1 integrin and their associated signaling pathways promoted cell proliferation, migration, invasion and survival in leading malignant phenotype formation [32]. β1 integrin is a kind of transmembrane receptor that communicates with a large number of downstream signaling molecules including integrin-linked kinase (ILK), Caveolin-1, or FAK to trigger survival pathway [33]. The downstream signaling alterations induced by β1 integrin are dependent on different cell types of cancer. For example, β1 integrin triggers the activation of FAK in contributing chemoresistance and radioresistance in pancreatic cancer and NSCLC [34,35]. Integrin-dependent activation of Wnt/β-catenin signaling can promote metastasis in ovarian cancer [36]. β1 integrin and ILK can trigger the activation of NF-κB to induce cell motility [14]. Conversely, malignant phenotype of tumor cell was inhibited in β1 integrin-depleted cells [14,37]. Therefore, these lines suggest that β1 integrin could be a prognostic marker of survival and therapeutic target in cancer treatment. Based on cell type dependency, identification of specificity of integrin-mediating downstream signaling in different types of cancer is an important issue for the development of therapeutic strategies and diagnostic tools.

Breast cancer is a heterogeneous disease. Seeking and developing a personalized therapy is an important issue in improving outcomes of clinical patients. Pre-clinical and clinical data have suggested that TNBC could be more sensitive to platinum-based chemotherapy in patients with defective homologous recombination repair. However, several randomized trials in evaluating platinum efficacy indicated conflicting results for long-term outcomes [38]. Therefore, it is worth to identify a predictive gene signature in TNBC treatment response. Previously, such evidence has also supported that increased β1 integrin is positively correlative with poor outcome in invasive breast cancer [31]. Interestingly, previous evidence demonstrated that expressions of ER and PR were not correlated with expression of β1-integrin in clinical outcomes [39]. Consistent with this finding, Klahan et al. colleagues combined microarray data from two databases and suggested that β1 integrin is a potential candidate biomarker of TNBC patients [40]. Therefore, we next investigated clarification of the role of β1 integrin in TNBC patients. In agreement with these findings, our clinical results also showed that high β1 integrin expression had significant high metastatic stage, significant high tumor recurrent rate, and significant low survival rate, compared to patients with low β1 integrin expression. In addition, the average disease-specific survival time in patients with high β1 integrin expression was significantly lower than that in patients with low β1 integrin expression. Our univariate analysis showed the overall survival was also significantly associated with β1 integrin expression, nodal stage, metastatic stage and tumor recurrence, and the multivariate Cox regression analysis also showed that β1 integrin expression was a significant independent predictor of overall survival. Therefore, our data strongly suggested that β1 integrin could be a potential prognostic marker of survival in TNBC patients. Therefore, we next focused on illustrating details of mechanism in β1 integrin-mediating malignant phenotype of TNBC.

The dissemination of cancer cells from the primary tumor, and their invasion in and out of blood vessels has been linked to EMT-related processes [41]. Thereby, we firstly tried to validate the role of β1 integrin in regulating TNBC cell migration, invasion and EMT. We used the MDA-MB-231, HS578T, and MDA-MB-468 cells as the models in this study. MDA-MB-231 and HS578T cells are classified as mesenchymal-like basal B-subtype and highly invasive whereas MDA-MB-468 cell is categorized as basal A-subtype and weakly invasive [42,43]. Figure 1 shows the western blot results indicating that MDA-MB-231 and HS578T cells had significantly higher β1 integrin expression compared to MDA-MB-468 and M10 cells. The MDA-MB-231 and HS578T cells also had higher migration compared to MDA-MB-468 cells (Figure 2). Knockdown of the β1 integrin expression in MDA-MB-231 and HS578T cells decreased the migration and invasion abilities, however, activation of the β1 integrin in MDA-MB-468 cell could increase the migration and invasion abilities. Besides, the western blot results also indicated that β1 integrin regulated several EMT-relative proteins in TNBC cells, including β-catenin, N-cadherin, E-cadherin and vimentin. This indicated that β1 integrin was involved in cell migration and invasion through regulating the expression of EMT-associated proteins in TNBC cells.

Besides, β1 integrin is associated with drug resistance in several cancer types [44,45,46]. For example, Huang et al. provided that blocking of β1 integrin could enhance cell response to lapatinib in overexpression of epidermal growth factor receptor breast cancer cells [26]. Park et al. reported that the β1 integrin inhibition dramatically increased the radiotherapy efficacy in a xenograft model [31]. Additionally, β1 integrin deficiency could attenuate malignant phenotype of bevacizumab-resistant cells [47]. In the present study, our results also demonstrated that β1 integrin expression was associated with phosphorylated levels of FAK and AKT. Silencing of β1 integrin increased cells response to cisplatin and decreased the activity of FAK and AKT in MDA-MB-231 and HS578T cells. Treatment of β1 integrin activator promoted the activity of FAK and AKT in MDA-MB-468 cells. More importantly, overexpression of FAK can reverse AKT activity and overcome the toxic effect of cisplatin in MDA-MB-231 cells, suggesting that FAK-AKT axis plays a role in β1 integrin-mediated defense system in counteracting cisplatin-induced apoptosis. It is worth noting that several preclinical trials have supported β1 integrin as promising chemotherapeutics in efficiently reducing tumor growth and metastasis in vivo [32]. Our finding, no doubt, provides a prompt therapeutic strategy in the treatment of TNBC.

In summary, we reported that high β1 integrin expression is correlated with low survival rates and advanced metastatic status in TNBC patients. β1 integrin also plays a regulatory role in TNBC cell migration, invasion, and EMT. Additionally, β1 integrin could regulate drug sensitivity in TNBC cells. Taken together, our study clearly indicates that β1 integrin could not only be a potential prognostic biomarker in TNBC patients, but targeting β1 integrin-FAK-AKT axis could be an effective therapeutic strategy in β1 integrin overexpressing TNBC cells as well. It has been showed that the level of β1 integrin is strictly regulated by miRNA [48,49]. Hereby, development of β1 integrin-associated microRNA may be helpful for therapeutic application in TNBC.

## 4. Materials and Methods

### 4.1. Cell Culture

TNBC cancer cells (MDA-MB-231, HS578T, MDA-MB-468) (ATCC, Manassas, VA, USA) and H184B5F5/M10 human mammary epithelial cell (BCRC, Hsinchu, Taiwan) were used in this study. Cells were maintained in Dulbecco’s modified Eagle’s medium (DMEM) and DMEM/F12 medium with 10% fetal bovine serum (FBS) (Hyclone Laboratories Inc., South Logan, UT, USA) and antibiotics at 37 °C in a 5% CO_2_ atmosphere. 

### 4.2. Reagents

Antibodies against β1 integrin (#9699, 1:1000), FAK (#3285, 1:1000), phospho-FAK(Y397) (#3283, 1:1000), Akt (#9272, 1:1000), phospho-Akt (S473) (#9271, 1:1000), Caspase 3 (#9662S, 1:1000), cleaved PARP (#9541, 1:1000), p44/42 MAPK (ERK1/2) (#9107, 1:1000), phospho-p44/42 MAPK (ERK1/2, Thr 202/Tyr 204) (#9101, 1:1000), Vimentin (#5741, 1:1000), N-Cadherin (#13116, 1:1000), β-Catenin (#8480, 1:1000), GAPDH (#2118, 1:5000) and E-Cadherin (#3195, 1:1000) were purchased from Cell Signaling Technology (Beverly, MA, USA). Mouse IgG1 negative control (400102, 10 μg/mL) was from Biolegend (San Diego, CA, USA). Anti-β1 integrin activating antibody (MAB1951Z (P4G11), 10 μg/mL) was obtained from Millipore Corporation (Temecula, CA, USA).

### 4.3. Immunoblots

Proteins extraction and immunoblotting were performed as previously described [33]. In brief, cells were sonicated in protein lysis buffer (M-PER^TM^ mammalian protein extraction buffer, Thermo Scientific, Rockford, IL, USA) and cellular debris was removed by centrifugation. Proteins were loaded on an SDS-polyacrylamide gel and transferred to nitrocellulose, and immunoblotting was performed by using the indicated antibodies.

### 4.4. β1 Integrin Short Interfering RNA and FAK Plasmid Transfection

Short interfering RNA (siRNA) for human β1 integrin (ITGB1, L-004506-00) and negative control (Lamin A/C, D-001050-01-05) were purchased from Dharmacon (Dharmacon Life Technologies, Cologne, Germany). MDA-MB-231 and HS578T cells were transfected with 100 nM nontargeting and specific siRNA using Lipofectamine 2000 and Opti-MEM medium according to the standard protocol provided by Invitrogen (Carlsbad, CA, USA). Constitutively active EGFP-FAK expression constructs were a gift obtained from Chen Hong-Chen’s lab (Graduate Institute of Biomedical Sciences, National Chung Hsing University, Taichung, Taiwan). β1 integrin silencing cells were transfected with the appropriate amount of expression construct and control empty vector using Lipofectamine 2000 and Opti-MEM medium as mention earlier.

### 4.5. Cell Proliferation Assay

1 × 10^4^ cells were plated in each well of a 24-well plate. After treated with different doses of cisplatin, cells were stained with MTT solution (Sigma, St. Louis, MO, USA) and incubated for 1–2 h. Finally, the formazan crystals were solubilized in 200μL DMSO (Sigma, St. Louis, MO, USA) and measured at 560 nm.

### 4.6. Cell Wound Scrape Assay

Cells of each group were cultured in 6-well plates. The confluent cell monolayer was made a wound area by a 200 μL pipette tip. Cells were then incubated in culture medium at 37 °C in a 5% CO_2_ incubator for 24 h. Images were captured at 0 and 24 h. The experiments were repeated in triplicate.

### 4.7. Cell Invasion Assay

Cell invasion assay were performed as previously described [40]. In brief, cells were seeded in the inserts placed in the upper chamber of transwell. 500 μL DMEM containing 10% FBS was plated in the bottom chamber. After 24 h incubation, cells were rinsed and stained with crystal violet solution (Sigma, St. Louis, MO, USA). The number of invading cells was resulted from triplicate cell invasion assays.

### 4.8. Specimens

Formalin-fixed, paraffin-embedded blocks of tissues from 67 TNBC patients were selected from the Department of Pathology, Kaohsiung Medical University Hospital, Kaohsiung, Taiwan. Institutional Review Board approval for using these human tissues in this study was given by the Research Ethics Committee of the Kaohsiung Medical Hospital (IRB: KMUHIRB-E(II)-20150086) on 22 June 2015.

### 4.9. Immunohistochemistry (IHC) Staining 

Blocks of tissue samples embedded in paraffin were sectioned into thicknesses of 4 μm. The samples were de-paraffinized, rehydrated, and then autoclaved at 121 °C for 9 min in pH 6.0 DAKO target retrieval solution (DAKO, Carpinteria, CA, USA) to induce antigen retrieval. In each section, endogenous peroxidase was blocked by incubation in 3% hydrogen peroxide (Sigma, St. Louis, MO, USA) for 10 min. Sections were incubated with β1 integrin and FAK (sc-558, Santa Cruz Biotechnology, Inc., Dallas, TX, USA) primary antibodies at room temperature for 1 h. The DAKO REAL Envision Detection kit (DAKO, Carpinteria, CA, USA) was then applied for 1 h. Finally, sections were incubated in 3’3-diaminobenzidine for 5 min, counterstained with Mayer’s Hematoxylin, and mounted. Negative controls were prepared by replacing the primary antibodies with non-immune serum. 

### 4.10. Scoring

β1 integrin expression in different patients was scored according to Yao et al. [39] in which sample was scored based on the intensity of signal (0, 1+, 2+, 3+) and the proportion of positive cells (0 ≤ 10%, 1 = 10%–25%, 2 = 25%–50%, 3 ≥ 50%). The staining index was calculated as the product of the intensity of signal and the proportion of positive cells. The score of image ranged from 0 to 9. Score ≤ 4 was defined as negative/low expression and score ≥ 6 was indicated as positive/high expression.

### 4.11. Statistical Analysis

Expression of β1 integrin in TNBC tissues as determined by immunohistochemical staining was compared and assessed by Chi-square test. To evaluate the use of β1 integrin for TNBC prognosis, survival curves were performed by Kaplan-Meier method. A Cox proportional hazards model was used to evaluate univariate comparisons of overall survival with clinicopathologic variables. Two-tailed Student’s *t* test was used to compare the difference of groups. *P* value less than 0.05 was considered statistically significant. All statistic analyses were performed using SPSS 19.0 software (IBM Corp., Armonk, NY, USA).

## Figures and Tables

**Table 1:** Association of β1 integrin and FAK expressions in breast tumor tissues.

Protein Expression		β1 Integrin, *n* (%)		*p*-Value
		High	Low	
	High	22 (32.83)	19 (28.36)	
FAK, *n* (%)				
	Low	7 (10.45)	19 (28.36)	*p* = 0.031

**Table 2:** Relationship between β1 integrin as a prognostic and predictive marker in triple negative breast cancer expression and clinicopathological characteristics of TNBC patients (*n* = 67).

Parameters	*n*	β1 Integrin, *n* (%)		*p*-Value
		Low	High	
Total	67	38 (56.72)	29 (43.28)	
Age, *n* (%)				0.0878
≤40 years	57	35 (92.11)	22 (75.86)	
>40 years	10	3 (7.89)	7 (24.14)	
Size, *n* (%)				0.4753
≤2.0 cm	24	15 (39.47)	9 (31.03)	
>2.0 cm	43	23 (60.53)	20 (68.97)	
Grade, *n* (%)				0.7530
I/II	24	13 (34.21)	11 (37.93)	
III	43	25 (65.79)	18 (62.07)	
Tumor stage, *n* (%)				0.2893
T1	28	18 (47.37)	10 (34.48)	
T2/T3	39	20 (52.63)	19 (65.52)	
Nodal stage, *n* (%)				0.0958
N0	40	26 (68.42)	14 (48.28)	
N1/N2/N3	27	12 (31.58)	15 (51.72)	
Metastatic stage, *n* (%)				0.0389 *
M0	48	31 (81.58)	17 (58.62)	
M1	19	7 (18.42)	12 (41.38)	
Tumor recurrent, *n* (%)				0.0380 *
Absent	61	37 (97.37)	24 (82.76)	
Present	6	1 (2.63)	5 (17.24)	
Survival status, *n* (%)				0.0040 *
Survival	45	31 (81.58)	14 (48.28)	
Death	22	7 (18.42)	15 (51.72)	

Statistical analysis was performed using the Chi-squared test. * Statistically significant (*p* < 0.05).

**Table 3:** Univariate and multivariate logistic analysis of clinicopathological independent prognostic factors for survival of breast cancer patients (*n* = 67).

Factors	Univariate		Multivariate	
	HR (95% CI)	*p*-Value	HR (95% CI)	*p*-Value
β1 integrin expression		0.0040 *		0.0476 *
Low	1.0		1.0	
High	3.805 (1.533–9.446)		2.772 (1.011–7.600)	
Age		0.8954		0.9776
≤40 years	1.0		1.0	
>40 years	0.921 (0.269–3.149)		0.981 (0.254–3.784)	
Grade		0.1240		0.1565
I/II	1.0		1.0	
III	2.190 (0.807–5.947)		2.286 (0.728–7.172)	
Nodal stage		<0.0001 *		0.0005 *
N0	1.0		1.0	
N1/N2/N3	12.304(3.634–41.656)		9.602 (2.666–34.586)	
Metastatic stage		0.0019 *		0.4788
M0	1.0		1.0	
M1	3.895 (1.653–9.178)		1.462 (0.511–4.186)	
Tumor recurrent		0.0436 *		0.8340
Absent	1.0		1.0	
Present	3.077 (1.033–9.165)		1.142 (0.329–3.969)	

Statistical analysis was performed using Cox multivariate analysis. * Statistically significant (*p* < 0.05).
